# Association between blood pressure and retinal arteriolar and venular diameters in Chinese early adolescent children, and whether the association has gender difference: a cross-sectional study

**DOI:** 10.1186/s12886-018-0799-x

**Published:** 2018-06-04

**Authors:** Yuan He, Shi-Ming Li, Meng-Tian Kang, Luo-Ru Liu, He Li, Shi-Fei Wei, An-Ran Ran, Ningli Wang, the Anyang Childhood Eye Study Group

**Affiliations:** 1 Beijing Tongren Eye Center, Beijing Tongren Hospital, Beijing Ophthalmology & Visual Science Key Lab, Beijing Institute of Ophthalmology, Capital Medical University, Beijing, China; 2 Anyang Eye Hospital, Anyang, Henan China

**Keywords:** Hypertension, Adolescents, Retinal arteriolar diameter, Retinal venular diameter, Blood pressure

## Abstract

**Background:**

To establish the independent association between blood pressure (BP) and retinal vascular caliber, especially the retinal venular caliber, in a population of 12-year-old Chinese children.

**Methods:**

We have examined 1501 students in the 7th grade with mean age of 12.7 years. A non-mydriatic fundus camera (Canon CR-2, Tokyo, Japan) was used to capture 45^0^ fundus images of the right eyes. Retinal vascular caliber was measured using a computer-based program (IVAN). BP was measured using an automated sphygmomanometer (HEM-907, Omron, Kyoto, Japan).

**Results:**

The mean retinal arteriolar caliber was 145.3 μm (95% confidence interval [CI], 110.6–189.6 μm) and the mean venular caliber was 212.7 μm (95% CI, 170.6–271.3 μm). After controlling for age, sex, axial length, BMI, waist, spherical equivalent, birth weight, gestational age and fellow retinal vessel caliber, children in the highest quartile of BP had significantly narrower retinal arteriolar caliber than those with lower quartiles (*P* for trend< 0.05). Each 10-mmHg increase in BP was associated with narrowing of the retinal arterioles by 3.00 μm (multivariable-adjusted *P* < 0.001), and the results were consist in three BP measurements. The association between BP measures and retinal venular caliber did not persist after adjusting for fellow arteriolar caliber. And there was no significant interaction between BP and sex, age, BMI, and birth status.

**Conclusions:**

In a large population of adolescent Chinese children, higher BP was found to be associated with narrower retinal arterioles, but not with retinal venules. Sex and other confounding factors had no effect on the relationship of BP and retinal vessel diameter.

## Background

Major component of the circulatory system is composed of the microcirculation, which plays an important role in maintaining cardiovascular health. There is a widespread influence of blood pressure (BP) on the structure and function of microcirculation system. Early in the late nineteenth century, Marcus Gunn had put forward the statement that there were associations between microvascular abnormalities and cardiovascular diseases [1].

The retina is a unique structure of the eyes, where the in vivo microcirculation can be directly visualized and monitored non-invasively. Retinal microcirculation shares the same anatomic architecture and physiological feature with other terminal organs elsewhere in the body [2]. These characteristics increase its utility as a tool to study the clinical performance of microvascular diseases. Recently, with the improvement of retinal imaging particularly the computer-assistant analysis techniques from digital retinal images [3], plenty of epidemiological studies in adult populations have displayed that abnormal changes in retinal vascular caliber (predominantly retinal arteriolar and venular caliber) are closely associated with some systemic vascular abnormalities such as cardiovascular risk factors [4], hypertension [5], coronary heart disease [6], risk of diabetes and stroke [7], cerebral infarcts and white matter lesions [8], and renal disease [9], independent of other risk factors.

Despite increasing data on the risk prediction of retinal vascular caliber measurement in different population-based studies, there had been still some controversial opinions on association between retinal vascular changes and BP, especially for the retinal venular changes. Understanding the impacts of BP and changes to the retinal microvasculature in persons with different background is an important aspect of the study on microcirculation disease. Children are generally free of many systemic conditions and eye diseases (such as glaucoma or diabetic retinopathy, etc.) that could bring about confounding effects on observed associations. High BP in children and adolescents is more and more common in western countries [10], and BP levels and prevalence of hypertension has increased dramatically among children and adolescents in China [11]. It is encouraging that there are some studies on the association of BP and retinal vessel caliber in children recently, but substantial data on children group are still needed to provide the reference data.

In this study, we investigated the independent association between blood pressure measures and changes to the retinal microvasculature in a relatively large population of 12-year-old Chinese children. This study also assessed the potential modifying influences of age, BMI, birth parameters, especially sex, on the associations between BP and retinal vessel caliber.

## Methods

### Study population

**Fig. 1:**
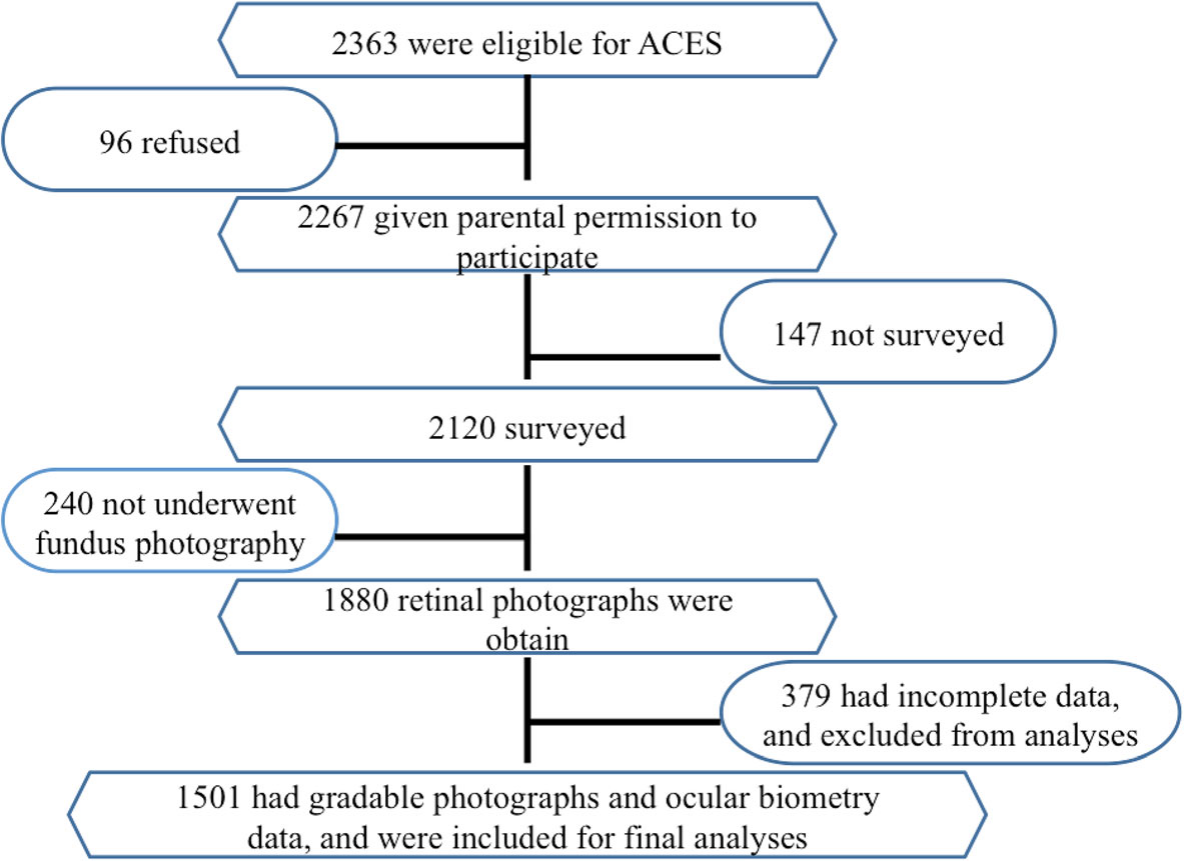
The flowchart of participants included in the present study

The Anyang Childhood Eye Study (ACES) is a school-based cohort study designed to observe the occurrence and development of myopia as well as other diseases in school children living in Anyang urban area, Henan Province, Central China. Detailed methodology of the study has been previously described [12]. In briefly, 1501 students in 7th grade average aged 12.7 years have been examined from October 2011 to December 2011. The flowchart of participants included in the present study was shown in Fig. 1. Ethics approval was obtained from the institutional review board of Beijing Tongren Hospital, Capital Medical University, and followed the tenets of the declaration of Helsinki. Informed written consent was obtained from at least one parent. Verbal assent was obtained from each child.

### Retinal photography and measurement of retinal vascular caliber

The children were examined at health examination station of the Anyang Eye Hosptial. A non-mydriatic fundus camera (Canon CR-2, Tokyo, Japan) was used to capture 45^0^ fundus images centering on optic disc and macular area of children’s right eyes by a well-trained operator [12]. Children with abnormal fundus images would also undergo left eye measurements, and we analyzed one picture for each child.

According to a standardized protocol described previously [3], the computer-imaging program (IVAN, University of Wisconsin, Madison, WI) was used to measure calibers of all retinal arterioles and venules located in zone 0.5 to 1 disc diameter from the optic disc margin (zone B). The program automatically combined vessel diameters from the six largest arterioles and six largest venules into a pair of indices. The central retinal arteriolar and venular equivalents (CRAE and CRVE) represent the average arteriolar and venular caliber for each eye, respectively. One grader masked to children’s identity and characteristics performed all measurements. Before starting the measurement, 50 randomly selected retinal images were repeatedly measured by the grader with an interval of 2 weeks. The reliability was high with intraclass correlation coefficients of 0.85 for arteriolar caliber and 0.97 for venular caliber.

### Blood pressure measurement

Blood pressure of children was measured in a seated position after 5 min of rest using an automated sphygmomanometer (HEM-907, Omron, Kyoto, Japan) with appropriate cuff size (bladder length ≈80% and width at least 40% of the arm circumference, covering the upper arm but not obscuring the antecubital fossa). Systolic and diastolic blood pressure (SBP and DBP, respectively) readings were taken. Two readings were taken 5 min apart and averaged for analysis. Mean arterial blood pressure (MABP) was computed as 2/3 of the diastolic plus 1/3 of the systolic value.

### Other measurements

Any abnormality of anterior segment (any abnormalities of the anterior segment of the eye, such as corneal leukoplakia, cataract, pupil abnormalities, iris anterior adhesion, etc.) was observed and recorded using a slit-lamp (YZ5J, 66 Vision Tech Co, Suzhou, China). Cycloplegic spherical equivalent refraction was measured using an autorefractor (HRK7000 A, Huvitz, Gunpo, South Korea) with three readings taken and averaged. An optical coherence biometry (IOL-master 1322–734, Carl Zeiss Meditec AG, Jena, Germany) was applied to evaluate the optical axial length (AL) value along the visual axis (line connecting the fixation point to the fovea, specifically from the anterior surface of the cornea to the retinal pigment epithelium layer of the fovea), with five repeated measurements taken and averaged. Height and weight were measured using an automatic and professional integrated set. Body mass index (BMI) was calculated as weight/height^2^ (kg/m^2^). Waist circumference was measured with a tape measure and was defined as the narrowest part of the student’s trunk. Birth information including gestational weeks, birth weight and birth length were collected by administrating questionnaires to the participating students’ parents.

### Statistical analysis

SAS (v9.3, SAS Institute Inc., Cary, NC, USA) was used to perform statistical analysis. BP was categorized into quartiles as well as being analyzed as a continuous variable (i.e. each 10 mmHg increase). The retinal arteriolar, venular calibers, and arteriolar to venular ratio (AVR) were compared across blood pressure quartiles based on three models, Model 1 was analyzed without any adjustment, Model 2 adjusted for multivariate variables (age, sex, axial length, BMI, waist, spherical equivalent, birth weight, and gestational age) and then Model 3 adjusted additionally for fellow retinal vessel diameter. The test of trend was determined by regarding quartiles of BP as continuous ordinal variables. Multiple linear regressions were used to estimate the absolute changes in retinal arteriolar and venular caliber for a 10-mmHg increase in SBP, DBP, and MABP. Potential modifiers were examined in stratified analyses of age, sex, BMI, and birth parameters. All probabilities quoted are two-sided, and a significant *P* value was defined as < 0.05.

## Results

Table 1 shows the study characteristics of the children included for crosss-sectional analyses. Compared with boys, girls had higher systolic and mean arterial blood pressure, higher waist and BMI, and had less myopia and longer axial length.

**Table 1:** Basic characteristics of the children included in the study

Characteristics	Male	Female	*P*
	(*n* = 792)	(*n* = 709)	
Age (year)	12.66 (0.50)	12.73 (0.49)	**0.004**
Spherical equivalent refraction (diopters)	−1.78 (2.08)	− 1.32 (2.02)	**< 0.001**
Axial length (mm)	23.93 (1.01)	24.35 (1.09)	**< 0.001**
Systolic blood pressure (mm Hg)	104.55 (10.87)	107.36 (10.35)	**< 0.001**
Diastolic blood pressure (mm Hg)	65.35 (7.57)	65.45 (7.21)	0.758
Mean arterial blood pressure (mm Hg)	78.42 (8.23)	79.41 (7.63)	**0.005**
BMI (kg/m^2^)	19.32 (3.22)	20.28 (3.91)	**< 0.001**
Waist (cm)	69.04 (7.84)	71.92 (10.14)	**< 0.001**

Data are mean (SD). *BMI* body mass index; Significant *p* values are bolded. Significant *p* values are bolded

Table 2 shows the mean retinal vascular caliber and AVR by quartiles of systolic, diastolic and mean arterial blood pressure in three different models. Children with highest quartile of BP were more likely to have narrower retinal arteriolar caliber than those in the lowest quartile after multivariable-adjustment (all *P* < 0.01), with a mean difference of 6–7 μm between the highest and lowest quartiles, and the results were consistent for three BP measurements. As for retinal venular diameter, in Model 1, children with higher BP had significantly narrower CRVE (*P* < 0.001 for trend for three BP measurements), and in Model 2, only children with higher SBP were found to have narrower CRVE than those with lower SBP (*P* = 0.038 for trend), however, this association did not persist after adjusting for fellow vessel caliber. In Model 1 and Model 2, children with higher BP quartiles had consistently and significantly narrower AVR (*P* < 0.001 for trend), and the results were consistent for three BP measurements.

**Table 2:** Mean retinal arteriolar diameter, retinal venular diameter, and AVR (mean and standard error) stratified by SBP, DBP and MABP

		n	Range (mm Hg)	Rentinal Arteriolar Diameter (μm)			Rentinal Venular Diameter (μm)			AVR	
				Model 1	Model 2	Model 3	Model 1	Model 2	Model 3	Model 1	Model 2
SBP	First quartile	375	79 to 100	149.02 ± 1.31	148.70 ± 0.62	147.91 ± 0.56	214.54 ± 0.63	214.91 ± 0.79	212.96 ± 0.71	0.696 ± 0.004	0.688 ± 0.007
	Second quartile	365	101 to 107	146.17 ± 0.62	146.19 ± 0.61	146.20 ± 0.54	213.16 ± 0.30	212.65 ± 0.77	212.12 ± 0.69	0.687 ± 0.002	0.685 ± 0.008
	Third quartile	373	107 to 113	144.24 ± 0.54	144.22 ± 0.60	144.36 ± 0.54	212.23 ± 0.20	212.30 ± 0.76	212.89 ± 0.68	0.681 ± 0.002	0.683 ± 0.008
	Fourth quartile	388	113 to 142	141.63 ± 1.36	141.99 ± 0.61	142.62 ± 0.55	210.97 ± 0.65	210.91 ± 0.78	212.76 ± 0.70	0.672 ± 0.004	0.680 ± 0.009
	**P for trend**			**< 0.001**	**0.001**	**< 0.001**	**< 0.001**	**0.038**	0.941	**< 0.001**	**< 0.0001**
DBP	First quartile	323	49 to 61	147.96 ± 2.50	149.14 ± 0.65	148.15 ± 0.58	214.04 ± 1.21	215.49 ± 0.82	213.29 ± 0.75	0.692 ± 0.007	0.687 ± 0.007
	Second quartile	407	61 to 65	145.95 ± 2.18	146.21 ± 0.57	146.19 ± 0.51	213.06 ± 1.05	212.73 ± 0.73	212.19 ± 0.65	0.686 ± 0.007	0.685 ± 0.008
	Third quartile	360	66 to 70	144.49 ± 2.20	144.53 ± 0.61	144.92 ± 0.55	212.35 ± 1.06	211.60 ± 0.77	212.01 ± 0.69	0.682 ± 0.007	0.683 ± 0.008
	Fourth quartile	411	70 to 90	142.99 ± 2.28	141.84 ± 0.58	142.31 ± 0.52	211.62 ± 1.10	211.36 ± 0.73	213.29 ± 0.66	0.677 ± 0.007	0.682 ± 0.010
	**P for trend**			**< 0.001**	**0.005**	**0.009**	**< 0.001**	0.078	0.970	**< 0.001**	**< 0.0001**
MABP	First quartile	370	60 to 75	148.62 ± 1.80	149.31 ± 0.61	148.24 ± 0.55	214.36 ± 0.87	215.75 ± 0.78	213.47 ± 0.71	0.695 ± 0.005	0.687 ± 0.007
	Second quartile	366	75 to 79	145.96 ± 1.55	145.81 ± 0.60	145.98 ± 0.54	213.07 ± 0.75	212.18 ± 0.77	211.87 ± 0.69	0.686 ± 0.005	0.685 ± 0.008
	Third quartile	400	79 to 84	144.28 ± 1.59	144.36 ± 0.58	144.56 ± 0.52	212.25 ± 0.77	212.12 ± 0.73	212.62 ± 0.66	0.681 ± 0.005	0.683 ± 0.008
	Fourth quartile	365	84 to 102	142.14 ± 1.84	141.50 ± 0.62	142.21 ± 0.56	211.22 ± 0.89	210.67 ± 0.79	212.78 ± 0.72	0.674 ± 0.005	0.680 ± 0.010
	**P for trend**			**< 0.001**	**0.011**	**0.004**	**< 0.001**	0.086	0.738	**< 0.001**	**< 0.0001**

Model 1:unadjusted model; Model 2: adjusted for age, gender, axial length, BMI, waist, spherical equivalent, birth weight and gestational age; Model 3: adjusted for fellow vessel diameter additionally. *SBP* systolic blood pressure, *DBP* diastolic blood pressure, *MABP* mean arterial blood pressure, *AVR* arteriolar to venular ratio. Significant *p* values are bolded

Table 3 shows the multivariable linear regression between retinal vascular caliber and BP. In model 1 and model 2, for each 10-mmHg increase in SBP, DBP and MABP, CRAE decreased by 3.07–4.40 μm (*P* < 0.001) and CRVE decreased by 1.47–2.69 μm (*P* < 0.001). In model 3 adjusted for fellow vessel caliber additionally, each 10-mmHg increase in BP was associated with 2.34–3.47 μm decrease in retinal arteriolar caliber (*P* < 0.001), but no significant change in CRVE (*p* > 0.42) was observed. AVR decreased by 0.010 to 0.014 for every 10-mmHg increase in BP in Model 1, Further adjustment for age, gender, axial length, BMI, waist, spherical equivalent, birth weight and gestational age had no impact on the magnitude of this effect (AVR reduction 0.007 to 0.012).

**Table 3:** Multivariate Linear Regression Models of Retinal Vascular Caliber and Blood Pressure

	Retinal arteriolar diameter (μm)		Retinal venular diameter (μm)		AVR	
	Mean (95%CI)	*P*	Mean (95%CI)	*P*	Mean (95%CI)	*P*
SBP						
Model 1	−3.23(− 3.96 to −2.52)	**< 0.001**	−1.57(− 2.50 to − 0.63)	**< 0.001**	−0.01 (− 0.013 to − 0.007)	**< 0.001**
Model 2	− 3.07 (− 3.79 to − 2.34)	**< 0.001**	− 2.06 (− 2.97 to − 1.15)	**< 0.001**	− 0.007(− 0.010 to − 0.004)	**< 0.001**
Model 3	−2.34 (− 3.00 to − 1.69)	**< 0.001**	− 0.34 (− 1.18 to 0.50)	0.428	–	–
DBP						
Model 1	−3.85(− 4.88 to −2.82)	**< 0.001**	− 1.47 (− 2.78 to − 0.15)	**< 0.001**	−0.013 (− 0.017 to − 0.009)	**< 0.001**
Model 2	− 4.02 (− 4.96 to − 3.08)	**< 0.001**	− 2.34 (− 3.53 to − 1.15)	**< 0.001**	− 0.011(− 0.015 to − 0.007)	**< 0.001**
Model 3	−3.20 (− 4.05 to − 2.35)	**< 0.001**	− 0.06 (− 1.16 to 1.03)	0.909	–	–
MABP						
Model 1	− 4.37(−5.35 to −3.39)	**< 0.001**	− 1.84 (− 3.11 to − 0.56)	**< 0.001**	−0.014 (− 0.019 to − 0.010)	**< 0.001**
Model 2	− 4.40 (− 5.34 to − 3.47)	**< 0.001**	−2.69 (− 3.88 to − 1.50)	**< 0.001**	− 0.012(− 0.016 to − 0.007)	**< 0.001**
Model 3	−3.47 (− 4.31 to − 2.62)	**< 0.001**	−0.21 (− 1.31 to 0.89)	0.712	–	–

Model 1:unadjusted model; Model 2: adjusted for age, gender, axial length, BMI, waist, spherical equivalent, birth weight and gestational age; Model 3: adjusted for fellow vessel diameter additionally. *SBP* systolic blood pressure, *DBP* diastolic blood pressure, *MABP* mean arterial blood pressure, *AVR* arteriolar to venular ratio. Significant *p* values are bolded

Subgroup analysis stratified by potential effect modifiers was presented in Tables 4, 5 and 6. Associations were consistent across subgroups stratified by age, sex, BMI, and birth parameters.

**Table 4:** Subgroup analysis stratified by potential effect modifiers of retinal Arteriolar Diameter with BP, stratified by potential modifiers

Potential Effect Modifiers			*n*	Retinal Arteriolar Diameter (μm)					
				Model 1	*p*	Model 2	*P*	Model 3	*P*
SBP	Age	10 + 11	70	−1.97 ± 1.66	0.238	−0.60 ± 1.79	0.736	−1.28 ± 1.54	0.411
		12	1122	−3.63 ± 0.43	**< 0.01**	− 3.31 ± 0.42	**<.001**	− 2.46 ± 0.39	**<.001**
		13 + 14 + 15	309	−2.6 ± 0.82	**< 0.01**	− 2.60 ± 0.84	**0.002**	−1.98 ± 0.73	**0.007**
	Sex	Male	792	−3.1 ± 0.52	**< 0.01**	− 3.41 ± 0.52	**<.001**	−2.57 ± 0.45	**<.001**
		Female	709	−3.05 ± 0.52	**< 0.01**	− 2.73 ± 0.52	**<.001**	−2.12 ± 0.49	**<.001**
	BMI	Upper 50%	750	−2.37 ± 0.57	**< 0.01**	−2.39 ± 0.5	**<.001**	−1.72 ± 0.49	**<.001**
		Lower 50%	751	−4.03 ± 0.55	**< 0.01**	−3.71 ± 0.52	**<.001**	−2.89 ± 0.46	**<.001**
	Birth weight	Upper 50%	778	−3.69 ± 0.53	**< 0.01**	−3.09 ± 0.53	**<.001**	−2.35 ± 0.48	**<.001**
		Lower 50%	723	−2.87 ± 0.51	**< 0.01**	−2.97 ± 0.52	**<.001**	−2.28 ± 0.47	**<.001**
	Gestational age	Term	1372	−3.27 ± 0.39	**< 0.01**	− 3.10 ± 0.39	**<.001**	−2.33 ± 0.35	**<.001**
		Preterm	129	−3.06 ± 1.09	**< 0.01**	−2.46 ± 1.12	**0.03**	−2.21 ± 1.02	**0.033**
DBP	Age	10 + 11	70	−1.84 ± 2.04	0.370	−0.78 ± 2.18	0.722	− 1.81 ± 1.88	0.342
		12	1122	−4.85 ± 0.62	**< 0.01**	−4.74 ± 0.56	**<.001**	−3.76 ± 0.51	**<.001**
		13 + 14 + 15	309	−1.35 ± 1.11	**< 0.01**	−2.27 ± 1.08	**0.037**	−1.36 ± 0.95	0.154
	Sex	Male	792	−4.2 ± 0.74	**< 0.01**	− 4.65 ± 0.69	**<.001**	−3.67 ± 0.60	**<.001**
		Female	709	−3.68 ± 0.72	**< 0.01**	−3.46 ± 0.67	**<.001**	−2.79 ± 0.62	**<.001**
	BMI	Upper 50%	750	−3.33 ± 0.75	**< 0.01**	−3.73 ± 0.68	**<.001**	−3.06 ± 0.62	**<.001**
		Lower 50%	751	−4 ± 0.75	**< 0.01**	−4.30 ± 0.68	**<.001**	−3.34 ± 0.60	**<.001**
	Birth weight	Upper 50%	778	−3.83 ± 0.73	**< 0.01**	−3.73 ± 0.66	**<.001**	−3.01 ± 0.59	**<.001**
		Lower 50%	723	−3.92 ± 0.75	**< 0.01**	−4.31 ± 0.70	**<.001**	−3.42 ± 0.64	**<.001**
	Gestational age	Term	1372	−3.89 ± 0.55	**< 0.01**	−3.95 ± 0.51	**<.001**	−3.10 ± 0.46	**<.001**
		Preterm	129	−3.32 ± 1.57	**< 0.01**	−4.35 ± 1.50	**0.005**	−3.69 ± 1.39	**0.009**
MABP	Age	10 + 11	70	−2.21 ± 2.03	0.280	−0.81 ± 2.17	0.709	−1.83 ± 1.88	0.334
		12	1122	−5.22 ± 0.58	**< 0.01**	−5.01 ± 0.56	**<.001**	−3.91 ± 0.51	**<.001**
		13 + 14 + 15	309	−2.41 ± 1.09	**< 0.01**	−3.00 ± 1.09	**0.006**	−2.04 ± 0.95	**0.034**
	Sex	Male	792	−4.40 ± 0.70	**< 0.01**	−4.91 ± 0.68	**<.001**	−3.81 ± 0.59	**<.001**
		Female	709	−4.21 ± 0.70	**< 0.01**	−3.89 ± 0.68	**<.001**	−3.12 ± 0.64	**<.001**
	BMI	Upper 50%	750	−3.58 ± 0.75	**< 0.01**	−3.88 ± 0.68	**<.001**	−3.06 ± 0.63	**<.001**
		Lower 50%	751	−4.90 ± 0.73	**< 0.01**	− 4.87 ± 0.68	**<.001**	−3.81 ± 0.60	**<.001**
	Birth weight	Upper 50%	778	−4.68 ± 0.71	**< 0.01**	−4.31 ± 0.68	**<.001**	−3.40 ± 0.61	**<.001**
		Lower 50%	723	−4.11 ± 0.70	**< 0.01**	−4.42 ± 0.68	**<.001**	−3.49 ± 0.62	**<.001**
	Gestational age	Term	1372	−4.40 ± 0.53	**< 0.01**	−4.38 ± 0.51	**<.001**	−3.39 ± 0.46	**<.001**
		Preterm	129	−3.98 ± 1.49	**< 0.01**	−4.23 ± 1.47	**0.005**	−3.66 ± 1.35	**0.008**

Model 1:unadjusted model; Model 2: adjusted for age, gender, axial length, BMI, waist, spherical equivalent, birth weight and gestational age; Model 3: adjusted for fellow vessel diameter additionally. *SBP* systolic blood pressure, *DBP* diastolic blood pressure, *MABP* mean arterial blood pressure. Term means pregnancy lasts longer than 37 weeks, and preterm represents that the duration of pregnancy is less than 37 weeks. Significant *p* values are bolded

**Table 5:** Subgroup analysis stratified by potential effect modifiers of retinal Venular Diameter with BP, stratified by potential modifiers

Potential Effect Modifiers			n	Retinal Venular Diameter (μm)					
				Model 1	*p*	Model 2	*P*	Model 3	*P*
SBP	Age	10 + 11	70	0.40 ± 2.16	0.855	1.43 ± 1.99	0.476	1.78 ± 1.71	0.302
		12	1122	−2.1 ± 0.55	**< 0.001**	−2.52 ± 0.54	<.001	−0.73 ± 0.50	0.148
		13 + 14 + 15	309	− 0.53 ± 1.08	0.622	−1.61 ± 1.06	0.129	0.02 ± 0.94	0.986
	Sex	Male	792	−1.12 ± 0.66	0.092	−2.03 ± 0.65	**0.002**	0.14 ± 0.57	0.809
		Female	709	−1.72 ± 0.69	**0.013**	−2.15 ± 0.68	**0.002**	−0.85 ± 0.64	0.186
	BMI	Upper 50%	750	−1.66 ± 0.75	**0.028**	−2.11 ± 0.69	**0.002**	−0.84 ± 0.64	0.186
		Lower 50%	751	−2.67 ± 0.69	**< 0.001**	−2.13 ± 0.64	**0.001**	0.06 ± 0.58	0.923
	Birth weight	Upper 50%	778	−1.97 ± 0.68	**0.004**	−2.05 ± 0.67	**0.002**	−0.27 ± 0.61	0.654
		Lower 50%	723	−1.19 ± 0.67	0.074	−2.06 ± 0.66	**0.002**	−0.43 ± 0.61	0.478
	Gestational age	Term	1372	−1.61 ± 0.5	**0.001**	−2.19 ± 0.49	**<.001**	−0.46 ± 0.45	0.309
		Preterm	129	−1.13 ± 1.55	0.467	−0.88 ± 1.64	0.594	0.62 ± 1.53	0.688
DBP	Age	10 + 11	70	2.59 ± 2.64	0.330	2.16 ± 2.42	0.376	2.62 ± 2.08	0.213
		12	1122	−2.42 ± 0.79	**0.002**	−2.91 ± 0.72	**<.001**	−0.31 ± 0.67	0.648
		13 + 14 + 15	309	0.3 ± 1.45	0.838	−2.33 ± 1.36	0.089	−0.92 ± 1.20	0.442
	Sex	Male	792	−1.11 ± 0.94	0.239	−2.38 ± 0.86	**0.006**	0.60 ± 0.76	0.433
		Female	709	−1.96 ± 0.96	**0.042**	−2.35 ± 0.86	**0.007**	−0.69 ± 0.82	0.4
	BMI	Upper 50%	750	−1.42 ± 0.99	0.151	−2.12 ± 0.89	**0.017**	−0.10 ± 0.82	0.907
		Lower 50%	751	−2.11 ± 0.95	**0.026**	−2.46 ± 0.84	**0.004**	0.07 ± 0.76	0.93
	Birth weight	Upper 50%	778	−1.47 ± 0.93	0.112	−1.98 ± 0.84	**0.018**	0.18 ± 0.76	0.811
		Lower 50%	723	−1.48 ± 0.98	0.132	−2.65 ± 0.90	**0.003**	−0.28 ± 0.83	0.733
	Gestational age	Term	1372	−1.59 ± 0.71	**0.024**	−2.37 ± 0.64	**<.001**	−0.15 ± 0.58	0.798
		Preterm	129	−0.02 ± 2.2	0.994	−2.43 ± 2.23	0.278	0.18 ± 2.12	0.931
MABP	Age	10 + 11	70	1.92 ± 2.65	0.469	2.13 ± 2.41	0.38	2.61 ± 2.07	0.213
		12	1122	−2.75 ± 0.76	**< 0.001**	−3.32 ± 0.71	**<.001**	− 0.59 ± 0.67	0.38
		13 + 14 + 15	309	− 0.12 ± 1.43	0.933	−2.48 ± 1.37	0.072	−0.62 ± 1.21	0.609
	Sex	Male	792	−1.34 ± 0.89	0.133	−2.68 ± 0.85	**0.002**	0.47 ± 0.75	0.535
		Female	709	−2.25 ± 0.94	**0.017**	−2.76 ± 0.88	**0.002**	−0.90 ± 0.84	0.284
	BMI	Upper 50%	750	−1.89 ± 0.99	0.056	−2.63 ± 0.89	**0.003**	−0.55 ± 0.83	0.513
		Lower 50%	751	−2.85 ± 0.93	**0.002**	−2.74 ± 0.83	**0.001**	0.13 ± 0.76	0.868
	Birth weight	Upper 50%	778	−2.13 ± 0.91	**0.020**	−2.52 ± 0.86	**0.003**	−0.02 ± 0.78	0.978
		Lower 50%	723	−1.55 ± 0.92	0.093	−2.80 ± 0.87	**0.001**	−0.38 ± 0.81	0.643
	Gestational age	Term	1372	−1.94 ± 0.68	**0.004**	−2.78 ± 0.64	**<.001**	−0.33 ± 0.59	0.577
		Preterm	129	−0.72 ± 2.13	0.736	−2.07 ± 2.18	0.345	0.49 ± 2.07	0.812

Model 1:unadjusted model; Model 2: adjusted for age, gender, axial length, BMI, waist, spherical equivalent, birth weight and gestational age; Model 3: adjusted for fellow vessel diameter additionally. *SBP* systolic blood pressure, *DBP* diastolic blood pressure, *MABP* mean arterial blood pressure. Term means pregnancy lasts longer than 37 weeks, and preterm represents that the duration of pregnancy is less than 37 weeks.Significant *p* values are bolded

**Table 6:** Subgroup analysis stratified by potential effect modifiers of AVR with BP, stratified by potential modifiers

Potential Effect Modifiers			*n*	AVR			
				Model 1	*p*	Model 2	*P*
SBP	Age	10 + 11	70	−0.010 ± 0.006	**0.118**	−0.007 ± 0.007	0.37
		12	1122	−0.010 ± 0.002	**<.001**	−0.007 ± 0.002	**<.001**
		13 + 14 + 15	309	−0.010 ± 0.003	**0.002**	−0.007 ± 0.004	0.05
	Sex	Male	792	−0.010 ± 0.002	**<.001**	−0.009 ± 0.002	**<.001**
		Female	709	−0.009 ± 0.006	**<.001**	−0.006 ± 0.002	**0.02**
	BMI	Upper 50%	750	−0.006 ± 0.002	**0.020**	−0.005 ± 0.002	0.06
		Lower 50%	751	−0.010 ± 0.002	**<.001**	−0.010 ± 0.002	**<.001**
	Birth weight	Upper 50%	778	−0.010 ± 0.002	**<.001**	−0.007 ± 0.002	**0.001**
		Lower 50%	723	−0.010 ± 0.002	**<.001**	−0.007 ± 0.002	**0.002**
	Gestational age	Term	1372	−0.010 ± 0.002	**<.001**	−0.007 ± 0.001	**<.001**
		Preterm	129	−0.010 ± 0.004	**0.035**	−0.008 ± 0.007	**0.015**
DBP	Age	10 + 11	70	−0.016 ± 0.008	**0.046**	−0.009 ± 0.009	0.314
		12	1122	−0.015 ± 0.002	**<.001**	−0.013 ± 0.002	**<.001**
		13 + 14 + 15	309	−0.007 ± 0.005	0.136	−0.003 ± 0.004	0.493
	Sex	Male	792	−0.016 ± 0.003	**<.001**	−0.014 ± 0.003	**<.001**
		Female	709	−0.011 ± 0.006	**0.003**	−0.009 ± 0.003	**0.009**
	BMI	Upper 50%	750	−0.011 ± 0.003	**<.001**	−0.011 ± 0.003	**<.001**
		Lower 50%	751	−0.012 ± 0.003	**<.001**	−0.012 ± 0.003	**<.001**
	Birth weight	Upper 50%	778	−0.013 ± 0.019	**<.001**	−0.011 ± 0.003	**<.001**
		Lower 50%	723	−0.013 ± 0.003	**<.001**	−0.012 ± 0.003	**<.001**
	Gestational age	Term	1372	−0.013 ± 0.002	**<.001**	−0.011 ± 0.002	**<.001**
		Preterm	129	−0.015 ± 0.007	**0.03**	−0.012 ± 0.007	0.137
MABP	Age	10 + 11	70	−0.016 ± 0.008	**0.048**	−0.009 ± 0.009	0.302
		12	1122	−0.015 ± 0.002	**<.001**	−0.013 ± 0.002	**<.001**
		13 + 14 + 15	309	−0.010 ± 0.005	**0.02**	−0.006 ± 0.005	0.192
	Sex	Male	792	−0.016 ± 0.002	**<.001**	−0.014 ± 0.002	**<.001**
		Female	709	−0.013 ± 0.003	**<.001**	−0.009 ± 0.003	**0.005**
	BMI	Upper 50%	750	−0.010 ± 0.003	**<.001**	−0.010 ± 0.003	**0.002**
		Lower 50%	751	−0.014 ± 0.003	**<.001**	−0.012 ± 0.003	**<.001**
	Birth weight	Upper 50%	778	−0.015 ± 0.003	**<.001**	−0.012 ± 0.003	**<.001**
		Lower 50%	723	−0.014 ± 0.003	**<.001**	−0.007 ± 0.007	**<.001**
	Gestational age	Term	1372	−0.014 ± 0.002	**<.001**	−0.012 ± 0.002	**<.001**
		Preterm	129	−0.016 ± 0.006	**0.018**	−0.012 ± 0.007	0.108

Model 1:unadjusted model; Model 2: adjusted for age, gender, axial length, BMI, waist, spherical equivalent, birth weight and gestational age. *SBP* systolic blood pressure, *DBP* diastolic blood pressure, *MABP* mean arterial blood pressure, *AVR* arteriolar to venular ratio. Term means pregnancy lasts longer than 37 weeks, and preterm represents that the duration of pregnancy is less than 37 weeks.Significant *p* values are bolded

It is worth noting that, there were no significant interactions between sex, and BP on retinal vessel diameters. The impact of BP on the diameters of retinal vessels showed no gender differences between boys and girls. In model 2, each 10-mmHg increase in BP was associated with 3.41–4.91 μm (*P* < 0.001) and 2.73–3.89 μm (*P* < 0.001) decrease in CRAE for boys and girls respectively, and in model 3, the decrease of CRAE reduced to 2.57–3.81 μm (*P* < 0.001) and 2.12–3.12 μm (*P* < 0.001) for boys and girls respectively (Table 4). And for CRVE, each 10-mmHg increase in BP resulted in 2.03–2.68 μm (*P* < 0.05) decrease for boys and 2.15–2.76 μm (*P* < 0.05) decrease for girls in model 2, but when CRAE was additionally adjusted in model 2, there is no significant association between BP and CRVE in either boys nor girls, which was consistent in three BP measurements (Table 5).

## Discussion

In this population of 12-year-old Chinese children, we found that increasing blood pressure was significantly associated with narrowing retinal arteriolar caliber and smaller AVR, but not with retinal venular caliber. After controlling for age, gender, axial length, BMI, waist, spherical equivalent, birth parameters and fellow retinal vessel, each 10-mmHg increase in BP was associated with an approximate 3~ 4 μm reduction in CRAE, and the changes were consistent of three BP measurements. The similar pattern and magnitude of change were also found in the relationship of BP with CRVE prior of taking confounding fellow arteriolar diameter into account, but after the fellow vessel were further adjusted, the change no longer had significant difference. And there was no significant interaction between BP and age, sex, BMI and birth status.

Both cross-sectional and longitudinal studies had provided substantial evidence that there is significant association between elevated blood pressure or hypertension and narrower central retinal arteriole caliber in adult populations [13–19]. However, there is conflicting evidence on retinal venular diameter as marker related to hypertension. Some studies [16, 18–22] suggested that retinal venular widening may be independently associated with risk of hypertension, others [15, 19, 23–25] had found no association, whereas some other researchers announced that both retinal venular and arteriolar caliber were inversely related to blood pressure, independent of age, gender, and smoking [26].

In addition, smaller retinal arteriolar caliber was also found to be associated with current alcohol consumption, greater body mass index and higher levels of total homocysteine [20], incident clinical stroke, carotid atherosclerosis, incident heart disease and cardiovascular mortality, as well as metabolic syndrome [13]. Larger venular calibers had been shown to be associated with atherosclerosis [27], inflammation [20–22, 27–30], stroke, cardiovascular mortality [13, 31], cigarette smoking [20, 27, 32, 33], and the metabolic syndrome (hyperglycemia, central obesity, and dyslipidemia) [16, 34]. These findings suggested that retinal venular widening may has pleiotropic associations with cardiovascular risk factors and diseases, and was not a specific biomarker for hypertension [35].

There have been some studies on relationship of blood pressure with retinal vessel calibers in children. Mitchell [36] reported that higher childhood blood pressure was associated with retinal arteriolar narrowing but not with retinal venular caliber in children aged 6–8 years. They found that each 10-mmHg increase in systolic blood pressure was associated with narrowing of retinal arterioles by 2.08 μm in Sydney children and 1.43 μm in Singapore children. In high school students aged 12.7 years, they found that elevated blood pressure was associated with narrower retinal arterioles, and also with wider retinal venules in boys, with each 10-mmHg increase in MABP associated with 2.02-mm decrease in retinal arteriolar caliber, and 2.19 μm increase in CRVE in boys (the Sydney Childhood Eye Study. SCES) [37]. In a later study on Singapore children aged 4~ 5 years, Li et al. [38] found that higher systolic blood pressure was associated with narrower retinal arterioles and wider retinal venules, with each 10-mmHg increase associated with 2.00 μm of retinal arteriolar narrowing and 2.51 μm of retina venular widening. In 2012, Hanssen examined 578 school children aged 11.1 ± 0.6 years from secondary schools in Germany and found that diastolic blood pressure was not only independently associated with arteriolar narrowing, but also with venular narrowing [39]. Imhof found that systolic and diastolic BP were associated with arteriolar narrowing in 391 Switzerland children with an average age of 7.3 years, but they failed to find the association between BP and venular diameter [40].

According to the results of the above studies, we found that just like the roles of retinal venular diameter play on the BP in adults, the relationship between retinal venular diameter and BP in the childhood population is still in controversy.

Unlike the SCES [37] (The subjects of this study were comparable in age to our research), we didn’t catch the finding that higher BP was associated with wider retinal venules in preadolescent boys. We speculated here that there are some possible reasons contributing to the discrepancy between the two results.

First, at present, there were some epidemiological studies on adolescent BP, but these studies had not reached the uniform conclusion related to the gender difference. Some studies showed a higher frequency of elevated BP in males than in females in children population based research [41], but these results differed from those obtained by Rosner B, whose study found that the prevalence of elevated BP significantly increased among girls (8.2% versus 12.6%; *P* = 0.007), but was only of borderline significance among boys (15.8% versus 19.2%; *P* = 0.057), after analyzing a population-based sample of 3248 children in National Health and Nutrition Examination Survey (NHANES) III (1988–1994) and 8388 children in continuous NHANES (1999–2008), aged 8 to 17 years [42]. The female subjects in our study were more frequently shown to have elevated BP compared to males. The SCES did not present whether there was a significant difference between girl and boy blood pressure. If the BP of two genders were basically similar, the difference of the retinal venular caliber maybe associated with other reasons.

Second, in the SCES, with regard to the mechanism underlying the conclusion that higher blood pressure was associated with wider retinal venules in boys, the author deduced that maybe it was because sex hormones had an protective effect on the retinal circulation, as a proportion of girls would have commenced puberty. But it was interesting that Zou found that in 76,869 Chinese girls, the rate of high blood pressure in menstruation group from 11 to 13 years was significant higher than that in the same age group of non-menstruation [43]. Similarly, there was conflicting evidence that hormone treatment could effectively reduce the risk of coronary heart disease, data from two large randomized clinical trials, the women’s health initiative (WHI) [44] and the heart estrogen and progestin replacement study (HERS) [45], found an increase in cardiovascular incidences in women taking hormone replacement therapy. In some adult population based studies, estrogen replacement therapy was found to be associated with narrower retinal arteriolar and venular calibers [46], independent of blood pressure and other vascular factors, but other researchers failed to found relationship between hormonal status in women and retinal vessel caliber [22]. Therefore, more and further researches were needed to acquire a greater depth of understanding on whether the hormone would have an effect on vessel diameter and would produce what kind of impact.

Third, the prevalence of child obesity is increasing rapidly worldwide, and the BMI may play an active role in the result of association of blood pressure and CRVE. Although a lot of literatures showed that the BMI was higher among boys than that of girls [47], Cole TJ reported that in population of 2–17 years of age, the prevalence of overweight is 25% in girls and 27% in boys, and obesity is 7 and 9% in males and females respectively [48]. In our studies, girls had a significantly higher BMI than boys (*P* < 0.0001). Obesity might influence the change of blood pressure by some mechanisms such as glomerular and tubular effects, and some of these mechanisms are sex dependent [49]. In the SCES research, they did not present the particular values of BMI for boys and girls. If the boys were more likely overweight just like that in other studies, they had a better chance to get wider venule than the same-aged girls. Although the BMI had been adjusted, high BMI might accompany by some possible physical abnormalities such as dyslipidemia, hyperglycemia and inflammation, which could result in wider retinal venules simultaneously.

Forth, lack of regular moderate-to-vigorous intensity physical activity is a well-known risk factor for cardiovascular disease, increasingly amount of studies have been focusing on the relationship between physical activity and retinal microcirculation and cardiovascular diseases [50]. Physical activity has been shown to be able to improve coronary endothelial function, reduce systemic blood pressure and improve early markers of atherosclerosis in pre-pubertal obese children. The association of higher levels of physical activity with better retinal vessel health have been demonstrated in adults as well as in children population [39, 51–53]. Before and during adolescence, girls usually undergo a lower level of physical exercise and greater decline in active physical activity than boys [54], which might explain the difference to some extent. Correspondingly, in our study, girls had higher waist circumference and BMI, which might result from insufficiency of physical activity compared with boys.

In addition to the reasons analyzed above, the association between blood pressure and retinal venular caliber might be affected by other factors such as smoking status [55], genetic and sex determinants, as well as ethnic differences. In summary, the association between BP and retinal venular caliber is a result of the interplay of many complicated reasons, maybe elevated blood pressure was associated with wider retinal venules in preadolescent boys, but due to the influence by comprehensive factors, the change was not significantly manifest in our study.

In the past, researchers had generally attributed a lower arteriolar-to-venular ratio (AVR) to generalized arteriolar narrowing and suggested that this ratio may provide information that would predict incident cardiovascular diseases. But with the advent of semi-automatic examination, it makes it possible to measure arteries and veins in retinal fundus separately. Since 2004, Ikram [27] and other researchers confirmed that elevated blood pressures were associated with smaller arteriolar diameters, but larger venular diameters were related to atherosclerosis, inflammation, and cholesterol levels. Hence, the idea that the AVR overall reflects generalized arteriolar narrowing should be reevaluated by taking into account the separate arteriolar and venular diameters. Therefore, many scholars suggested that arteriolar and venular diameters should be examined separately, especially in etiologic research [23]. In our study, we found that increasing blood pressure was significantly associated with narrowing retinal arteriolar caliber and smaller AVR, but not with retinal venular caliber.

Our results once again stressed the necessity of additional adjustment of concomitant vessels. We found a relationship between higher SBP and smaller CRVE, however, when CRAE was added to the final multivariate-adjusted model (model 3), the relationship between SBP and CRVE became nonsignificant, and further adjustment of the caliber of the CRVE diminished the reduction magnitude of CRAE when BP increased, suggesting the possibility of a confounding effect of fellow vessel caliber on this association. A significant association between narrower venular caliber and hypertension was initially reported in the Rotterdam Eye Study [23], but this result was diminished after additional adjustment with retinal arteriolar caliber, and the same conclusion was obtained by Myers [22]. The difference of the results illustrated the importance of correcting concomitant vessels.

Strengths of this study include its random cluster sample of a large number of representative healthy schoolchildren. The samples were free of influences from systemic disease processes or eye diseases on retinal vessel measurements. We also used a previously validated standardized protocol of quantitative retinal imaging program for retinal vessel measurement. However, some potential limitations of our study demand consideration. First, the study design is cross-sectional and does not provide temporal information on the associations. Second, the possible selection bias giving rise from the exclusion of students by ineligibility and ungradable retinal photographs may play a part on the real association between BP and retinal vessel diameters. Finally, we failed to acquire further information from our samples such as smoking status, family history, blood lipid levels, blood glucose, which may have an impact on the results.

In conclusion, this study shows that in population of 12-year-old Chinese children, increasing blood pressure was significantly associated with narrower retinal arteriolar caliber but not with retinal venular caliber, and possible confounding factors such as sex et al. had no effect on the relationship between BP and retinal vessel diameters. This finding provided further insight into the relationship of elevated BP on the microcirculation that occurs in early life. The association of wider retinal venular caliber and hypertension has not yet been consistently found, which should remain one of our highest research priorities.

## Abbreviations

BMI: Body mass index
BP: Blood pressure
CRAE: Central retinal arteriolar equivalents
CRVE: Central retinal venular equivalents
DBP: Diastolic blood pressure
MABP: Mean arterial blood pressure
SBP: Systolic blood pressure
